# Analysis of the Relationship Between Enguri Large Dam Monitoring Entropic Features

**DOI:** 10.3390/e27040413

**Published:** 2025-04-11

**Authors:** Tamaz Chelidze, Teimuraz Matcharashvili, Aleksandre Sborshchikovi, Ekaterine Mepharidze, Dimitri Tepnadze, Levan Laliashvili

**Affiliations:** Mikheil Nodia Institute of Geophysics, Ivane Javakhishvili Tbilisi State University, Tbilisi 0160, Georgia; tamaz.chelidze@gmail.com (T.C.); matcharashvili@gtu.ge (T.M.); a.sborshchikov@gmail.com (A.S.); dimitritefnadze7@gmail.com (D.T.); laliashvili.levan@gmail.com (L.L.)

**Keywords:** Enguri dam foundation displacement, water level variation in reservoir, linear and nonlinear analysis, Shannon and Tsallis entropy

## Abstract

In this research, the results of the analysis of Enguri Large Dam (West Georgia) monitoring features, such as foundation displacement data and water level (WL) variation in the reservoir, were investigated. A statistical approach based on calculating time series helps us determine the research area’s dynamic picture. In this article, we have used various nonlinear analysis methods. Nonlinear dynamics of deformation and filling/reloading near grand dams reflect the complexity of the mentioned time series, connected with the natural agents (regional and local geodynamics), which were presented even before dam erection, and the effects of the water level variation in the reservoir. Both these effects are documented by observations from 1974 to 2024 at the Enguri Large Dam. Modern linear and nonlinear primarily data analysis techniques will be used for analysis of monitoring characteristics of the Enguri Large Dam: Kullback–Leibler divergence, mutual information, Shannon entropy, and Tsallis entropy. The obtained data on the dynamics of deformation and filling/reloading near a large dam can be used for the assessment of the possible risks connected with abrupt changes in the routine dynamics of construction.

## 1. Introduction

In the 1970s, in the west of Georgia, construction of the 271 m high Enguri arch dam, which still remains one of the highest (in its class) dams in the world, was started. The Enguri arch dam is part of the Enguri Hydro Power Plant (HPP) system located in the Enguri Gorge, Georgia. Since the start of construction, multi-disciplinary geodynamical–geophysical monitoring was organized in the dam area [1,2,3,4,5]. The geological survey documented that the branch fault of the large, active Ingirishi fault crossed the right wing of the Enguri dam foundation. At the same time, it is known that the presence of an active (or potentially active) fault in a large dam foundation is a serious threat to dam safety. As such, the monitoring of the fault zone started well before the beginning of dam construction and HPP reservoir filling. Nowadays, we have unique databases of the deformation of the dam foundation, as well as water level variations in the reservoir. Some of these data sets are already published in scientific periodicals [6,7,8,9,10]. In the present research, we aimed to focus on the dam foundation displacement data sets in the period from 1974 to 2024 and the corresponding variations of Shannon and Tsallis entropies.

In the lower part of the station, a 22.5 m long quartz extensometer (deformograph) has been installed and has been operating on the right bank of the Enguri River fault since 1974. It continuously records the relative horizontal displacements of the blocks along the fault propagation direction using the photo-optical registration method. It is also worth noting that in 2014, a laser registration and telemetry system was installed on the strainmeter, as a result of which we automatically receive observation material via the Internet in Tbilisi at an hourly rate. So, currently, the registration of deformation processes occurring on the fault block is being carried out by a strainmeter using the laser registration method, the material of which (along with water level change and precipitation (rain) data) is automatically transmitted to Tbilisi every hour for general analysis, as we mentioned.

The observation of ongoing processes at the base of the Enguri Dam was started in 1974. In the moments that the reservoir was filled, we observed movement at the base (deformation), which became the subject of very interesting research.

Depending on the season, the water level in the Enguri Dam reservoir varies by 100 m, which means that the Enguri reservoir can activate additional quasiperiodic strains to the existing (slow) tectonic strains [11,12,13,14,15].

In the past years, papers have been published on new, nonlinear dynamics analysis and applications to dams. New, nonlinear dynamics analysis and applications aimed to conduct seismic analysis of concrete gravity dam nonlinear behavior are considered in the work [11], where authors investigate dam failure due to loss of shear strength and the existence of discontinuity within the foundation. Complex dynamics of fault zone deformation under large dams at various time scales is considered in [1]. In the papers, a time series of nearly 40 years of fault zone strain which crosses the foundation of the Enguri high arc dam was analyzed. The time series of fault strain reflects the summary effect of tectonic, environmental, and man-made impact. These contributions to the geomechanics of geo-energy are the first applications of complexity theory methods to the analysis of fault zone monitoring time series. The study [12] investigates the stability of an artificial dam used in an underground reservoir in a coal mine under periodic weighting imposed by overlying rock strata. The mechanical properties of constant-amplitude cyclic loading and unloading of artificial dam assemblies in abandoned mine reservoirs were studied.

In this work, we apply new methods of complexity analysis to assess, quantitatively, the correlation between water level variations and the deformation of the dam foundation. The research includes various important tasks from different scientific fields. It will be performed for the Enguri Dam’s feature datasets (displacement, water level). They have a significant advantage in terms of technical progress. The results will determine the entropic relationship between the strain characteristics (deformation of the dam foundation) of the Enguri Dam and water level variations.

## 2. Materials and Methods

In general, the main objective of a clustering algorithm is to compare classes (or clusters) and to group them using a divergence measure as a metric of similarity or dissimilarity between them [16,17,18,19,20]. In this work, we will introduce the results of traditional and modern, linear and nonlinear data analysis techniques such as Kullback–Leibler divergence, mutual information, Shannon entropy, and Tsallis entropy [21,22,23]. We chose these methods as they are applicable to non-extensive cases.

In the common parlance, similarity (or dissimilarity) is assessed by a number that measures how distinct data sets are ordered. To measure the statistical “distance” between the distributions p and q, for a given random variable x, it is common to use the Kullback–Leibler divergence [24].

In mathematical statistics, the Kullback–Leibler (KLD or DKL) divergence (also called relative entropy) is a type of statistical distance: a measure of how much a model probability distribution q is different from a true probability p.

(1) $$D_{KL}=\sum_{k\in x}^{n}p(x)\log_{2}\frac{p(x)}{q(x)}$$

As we know, the relative entropy must be a non-negative real number. It is equal to 0 only if the two distributions in question are identical. It has diverse applications, both theoretical, such as characterizing the relative (Shannon) entropy in information systems, randomness in continuous time series, and information gain when comparing statistical models of inference, as well as practical, such as applied statistics, fluid mechanics, neuroscience, bioinformatics, and machine learning.

In other words, the KL Divergence represents a measure of the degree of difficulty in discriminating between classes (the larger the divergence, the greater the separability between the classes).

As already mentioned, the use of distance measures in statistics is of fundamental importance in solving different practical problems, such as hypothesis testing, goodness of fit tests, etc., and as it was already underlined, especially for solving classification tasks. The main objective of this research was to represent the classification of compared groups, keeping in mind the features of the corresponding time series.

Shannon’s theory defines a data communication system composed of three elements: a source of data, a communication channel, and a receiver. The “fundamental problem of communication”—as expressed by Shannon—is for the receiver to be able to identify what data were generated by the source based on the signal received through the channel. Shannon considered various ways to encode, compress, and transmit messages from a data source, and proved in his source coding theorem that entropy represents an absolute mathematical limit on how well data from the source can be losslessly compressed onto a perfectly noiseless channel.

From the last research, it was shown that many complex systems do not always obey the classic Boltzmann–Gibbs–Shannon approach. In such systems, the probability of occurrence of different microstates is correlated; the distribution of events in such systems obey a power law due to long-range interactions. Tsallis suggested a generalized approach, non-extensive statistical mechanics (NESM), in which interactions among the elements of a system at all lengths are taken into account. Nowadays, we can see in various publications that the Tsallis entropy helps to build a new statistical mechanic, which is very useful for the description of fractures from the laboratory scale to a global seismic process. Tsallis entropy $S_{T}$ calculation is an often-used method for different complex time-series analysis. As we know, subsystems of real-world systems are not independent, and thus the Boltzmann–Gibbs–Shannon condition of extensivity is not satisfied. Consequently, long- and short-range correlations in such systems cannot be regarded as negligible at all scales. Thus, we face a non-extensive case, for which Tsallis introduced a special entropic expression:

(2) $$S_{T}=\frac{k}{q-1}(1-\sum_{i=1}^{\Omega }p_{i}^{q})$$

where k is Boltzmann’s constant and Ω is the total number of accessible i microstates of the system with a probability of occupation $p_{i}$. The number q, which characterizes the degree of non-extensivity of the system, is an entropic index. The Tsallis entropy, which quantifies the dynamic changes in the complexity of the system, is lower for the cases that are characterized by a lower complexity (are less random).

Entropy is the uncertainty of a single random variable. We can define conditional entropy H(X|Y)*,* which is the entropy of a random variable conditional on the knowledge of another random variable. The reduction in uncertainty due to another random variable is called mutual information. For two random variables, in our case, displacement (X (in formula x)) and water level variations (Y(in formula y)), this reduction is the mutual information MI (I).

(3) $$\;\;\;\;\;\;\;I\left(X;Y\right)=H\left(X\right)-H\left(X|Y\right)=\sum_{x,y}p\left(x,y\right)log\frac{p(x,y)}{p\left(x\right)p(y)}$$

As we know, the mutual information I(X;Y) is used for measuring the dependence between the two random variables. It is symmetric in X and Y, always nonnegative, and is equal to zero if, and only if, X and Y are independent.

Mutual information is a fundamental quantity for measuring the relationship between random variables. In data science, it has found applications in a wide range of domains, including biomedical sciences, blind source separation (BSS, e.g., independent component analysis), information bottleneck, feature selection, and causality. Mutual information is a Shannon entropy-based measure of dependence between random variables. The mutual information between X and Y can be understood as the decrease in the uncertainty in X given Y.

## 3. Results

**Figure 1:**
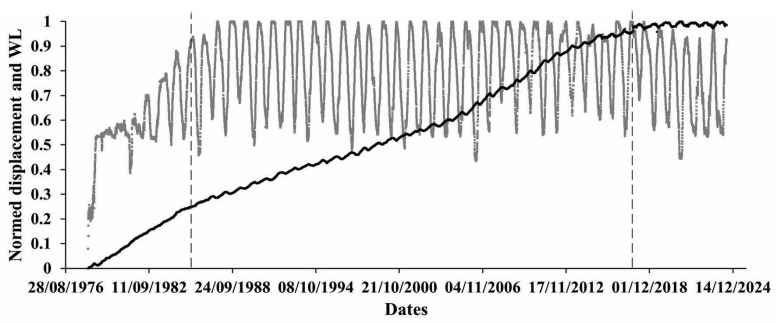
Normed displacement (black) and water level variations (gray). Here, and in following figures, the left vertical broken line marks the start of the regular WL regime and the right one the stop of the slow-component impact.

Previous studies have shown that tectonic crustal displacement beneath the high dam and reservoir area had increased (see the left side of Figure 1, bold black curve) prior to water impoundment in 1987 and continued to increase with more and more pronounced superimposed quasiperiodic oscillations under the influence of water level since 1987. Since then, the slow component of the crust displacement has remained basically unchanged, except for some variations in 2004. It was logical to assume that such a displacement mode will continue for a considerably long time. However, since about 2018, the evolution of the slow displacement component slowed down and later stopped, though water level variation in the reservoir continued, and the quasiperiodic component was present in the recording. This looked quite unexpected. Among the possible causes of such changes, the most obvious and logical one is the mode of influence of two main forces affecting the strain state of the large Enguri water reservoir: (1) the influence of tectonic force impact and (2) periodic variation of water masses in the large Enguri water reservoir. Of these two factors, the latter is well controlled and a corresponding data set of water level variation was available for us in the context of long-term observations related to Enguri Dam safety control activities. This inspired us to carry out a special analysis on entropy variation due to both factors: water level variation and the natural displacement of the Earth’s crust beneath a large dam foundation.

In Figure 1, the normed displacement and water level time series are presented. We applied the 0–1 normalization procedure to both time series due to the large difference in the displacement measurement results in the period of observation:

X=(x−min)/(max−min),

where x is the original sample data value and min and max are the minimum and the maximum values of measured data.

It is most noticeable that the evolution of displacement is characterized by an increasing trend from the time of observation to about 2015.

**Figure 2:**
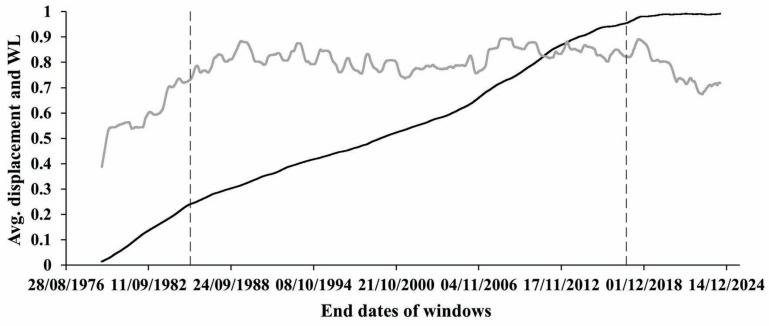
Normed displacement (black) and water level variation (gray) averaged in 365 data windows shifted by 1 data point.

At the same time, knowing that the main cycle in the processes of both water level variation and displacement evolution is one year, we decided to focus on the time series compiled from averaged data over one-year windows for both considered processes (see Figure 2). Consequently, in further analysis of different characteristics of targeted processes, we used windows of one-year lengths, which were shifted by 1 data point (1 day).

In Figure 1, and in following Figures, the left vertical line marks the start of the regular cycle of deformation according to reservoir load–unload cycle, and the right one marks the disappearance of the slow component of the fault motion.

**Figure 3:**
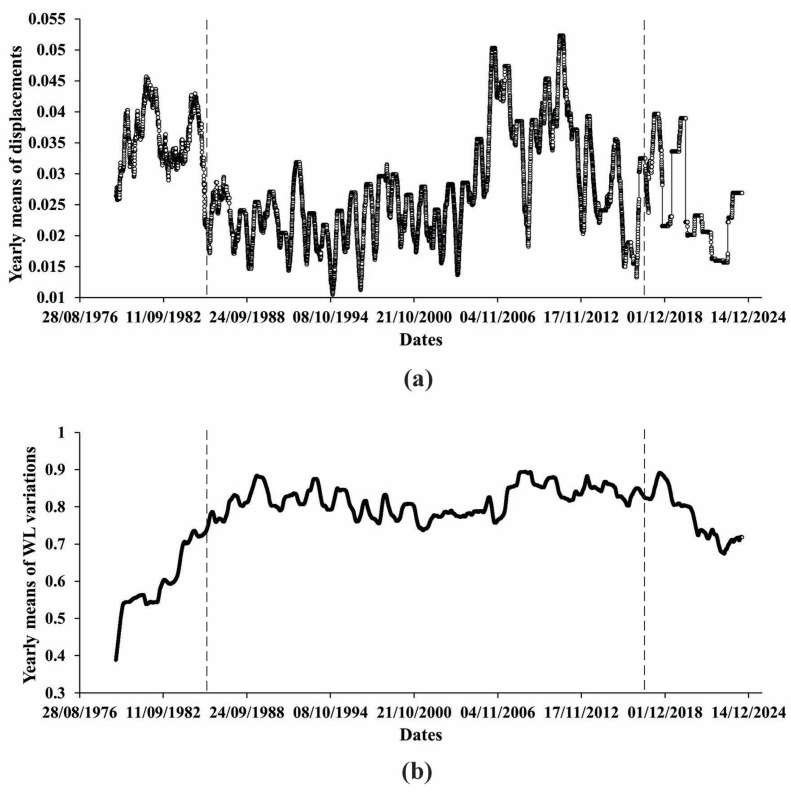
Differences between max and min values of mean yearly (**a**) displacements and (**b**) water level variation.

So far, as we are mainly interested in the character of the Earth’s crust displacement process beneath the high arch dam for a period of continuous observation, we preferred to focus on extreme changes of the displacement process on a yearly basis. In Figure 1, we see that under the influence of the water level change in the reservoir, the displacement process undergoes small but still clearly visible stair-like changes on the daily scale imposed on the abovementioned increasing trend of displacement evolution. On a yearly scale, these changes are not clearly visible in Figure 2, where mean values of displacements (black curve) are presented. At the same time, in Figure 3a, where we show the differences between the max and min values of the yearly variation of displacements, we see regular quasiperiodic changes that are obviously related to the water level variations. It deserves to be mentioned that these changes look most regular between 1987 and 2005.

Based on these data sets, in Figure 3b, we analyzed the variation of the extent of regularity and the functional relationship between the differences in the max and min values of yearly means of displacements and yearly mean values of water level. We started with the analysis of the regularity of the displacement process of the Earth’s crust beneath the Enguri dam from 1978 to 2024. As we see in Figure 3, there are three most noticeable parts of the displacement behavior for the analyzed period: 1. an almost linear increase; 2. quasiperiodic variations, and 3. a period of decreased displacement. These periods are not as clear as in Figure 1 but can still be distinguished.

**Figure 4:**
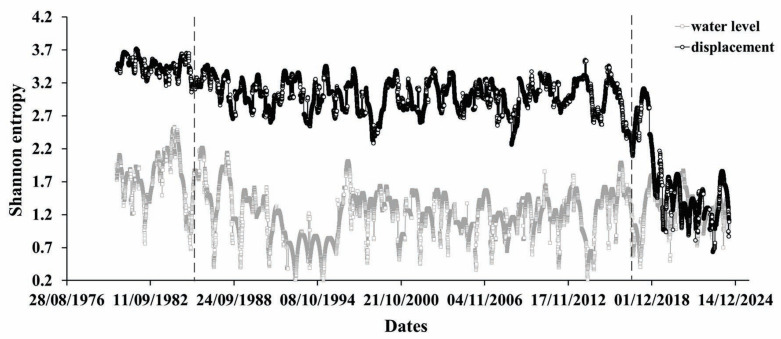
Shannon entropy calculated for 365 consecutive data windows of normed displacements (black) shifted by 1 data point. Daily water level variation (gray) is shown. There, Shannon entropy is not periodic, with 1 year WL variations until 1976.

According to Figure 4, (upper curve) the entire process of differences between the max and min yearly displacement evolution reveals the variable extent of regularity in the considered 365 data windows, starting from the irregular (largest Shannon entropy) period of changes that occurred at a yearly scale (windows shifted by one day), under the influence of flooding of the future reservoir area, since the start of the water level periodic variation process appears to have shifted slightly towards regularity for more than 10 years. Since about 2014, the displacement process has become much more regular compared to earlier periods of observation (Figure 4).

**Figure 5:**
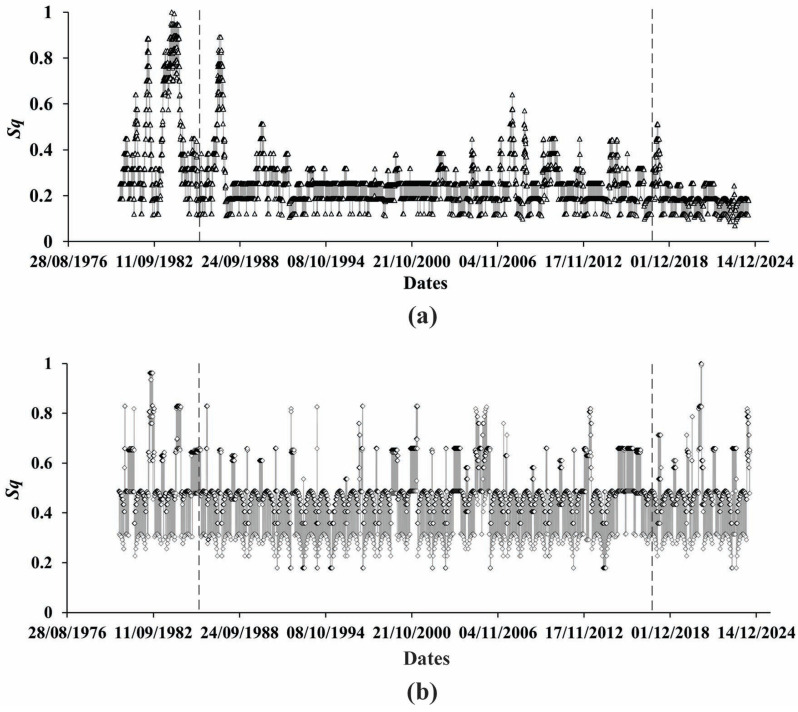
Tsallis entropy calculation for (**a**) differences between max and min values of mean yearly displacements (triangles points) and (**b**) mean values of water level yearly variation (rhombus points).

**Figure 6:**
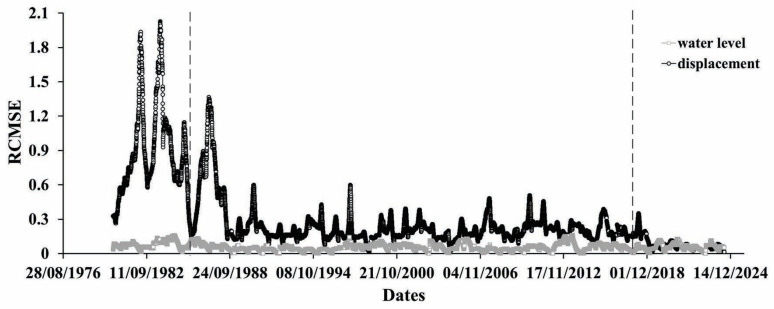
Shannon entropy calculation for differences between max and min values of mean yearly displacements (black) and mean values of water level yearly variation (gray).

In principle, the same conclusion can be drawn from Figure 5 and Figure 6. Indeed, the results of both the Tsallis entropy and refined composite multiscale entropy (RCMSE) calculations point to the highly disregulated process of differences between the extremes of yearly displacements becoming much more regular at the end of the observation period, where the tectonic component of strain practically disappears. The clear reactions of the dam foundation to the three characteristic periods in the strain dynamics (periods of natural state, combined action of tectonic and water load forces, and disappearance of tectonic component) are reflected in the changes of the Tsallis and Shannon entropies.

Figure 6, for RCMSE, seems to be the most interesting figure, as in it, all three characteristic stages of the fault strain evolution are very clearly represented, especially the last one, where, besides the absence of the very slow component of strain, a clear decay of the amplitude of yearly variations is visible. That can mean that, besides cutting out of the “secular” component of deformation, the significant reduction of the yearly strain variations due to loading/unloading of the lake also takes place.

**Figure 7:**
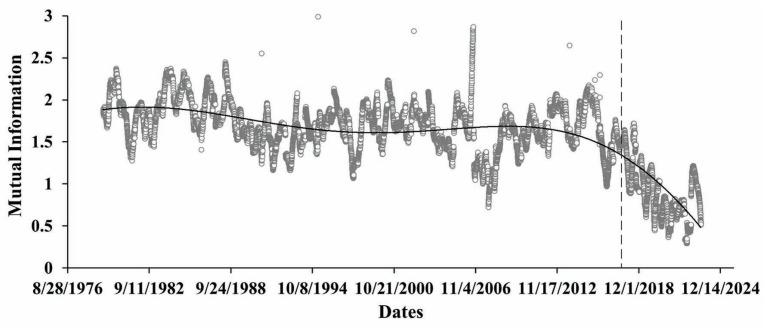
Mutual information for differences between max and mean yearly displacements and water level yearly mean variations calculated in windows of 365 days, shifted by 1 day (circles with trend line).

**Figure 8:**
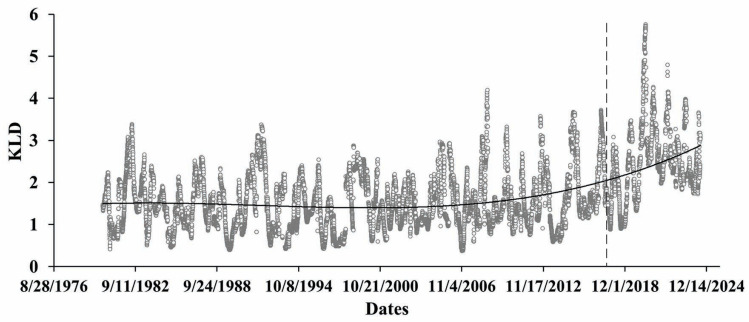
Kullback–Leibler divergence for differences between max and mean yearly displacements and water level yearly mean variations calculated in windows of 365 days, shifted by 1 day (circles with trend line).

We also present below the results of mutual information—MI (Figure 7), and Kullback–Leibler divergence calculations (Figure 8), aimed to assess the functional relationship of differences in the yearly extremal displacements and the yearly means of water level variation. Mutual information calculations show a clear decrease in the last period, where the tectonic component practically is absent.

After this, we proceeded to conduct similarity testing of displacement of the Earth’s crust and reservoir water level variations using KLD metrics. In Figure 8, we see that, like MI testing, dissimilar (KLD>1) processes of displacement and water level variation become practically similar in the same period since about 2018. Results from both figures point to the essential changes in the character of functional relationships over the analyzed period. Most noticeable is that starting from 2018 to the present day, the functional relationship between differences in displacement and water level variation on a yearly scale gradually decreased. It needs to be underlined that in this period, water level variation still remained mostly quasiperiodic, though the effect of the slow (tectonic) component seems to be decreasing, tending to negligence.

All presented data show that the trend component, with the slow strain rate, was recorded even before dam construction and lake filling (Figure 1). It is due to the long-term strain component created by a slow regional tectonic stress action. Thus, the fault zone deformation reflects a joint influence of local tectonics and man-made engineering stresses. In the last five years, the strain rate of the slow quasiperiodic component has abruptly dropped to zero. The change of the strain regime may be connected with either the final stabilization of the fault or a temporary braking of fault motion by a strong asperity on the fault interface. The brake of asperity can lead to earthquakes of magnitudes M3–4, which is not dangerous for the dam structure.

The entropic characteristics of the yearly variation component of the fault strain also depends on the relative amplitudes of the slow and periodic components; the Shannon entropy, Tsallis entropy, refined composite multiscale entropy, mutual information, and Kullback–Leibler divergence all show clear changes in the last six years, probably related to the decrease in the slow component of strain caused by asperity, braking the fault displacement. The strain rate on the fault in the period of 1974–2019 varied between 250 and 150 µm/year, but in recent years (2019–2023), the strain rate has abruptly dropped to zero. This change in the strain regime, both in the slow and early components of strain, may be connected with either the final stabilization of the fault or a temporary braking of fault motion caused by a strong asperity on the fault interface, which can lead to dynamical discharge of the accumulated strain. In recent years (2019–2023), the slow component of the strain rate also abruptly fell to zero. This change in the strain regime can be connected with either the final stabilization of the fault or a temporary full braking of fault motion by strong asperities on the fault interface. In this case, the stress on the fault will build up until it attains the critical stress value necessary for overcoming the resistance of asperities in a dynamic manner. The dynamic discharge of the accumulated energy on the small fault can generate an earthquake in the range of M3–4, which is not dangerous for the dam structure.

## 4. Conclusions

According to the analysis of the dynamics of tectonic displacement and water level variations over the considered period, they have undergone noticeable, but not similar, changes. Despite some quantitative changes, it can be said that the character of the dynamics of water level yearly mean variation, in general, has practically not changed since the period of regular water impoundment to the present day. At the same time, the behavior of differences between the extremes of yearly displacements underwent essential changes at the end of the observation period, which started in about 2017. Consequently, the functional relationship between water level variation and displacement began to decrease in a slow mode. As we can see from the same data, the start of water regulation in 1987 is evident. The entropic values reflect yearly water level variations. In some figures, the left vertical line marks the start of a regular cycle of deformation according to the reservoir load–unload cycle and the right vertical line marks the disappearance of the slow (tectonic) component of the fault motion, which can point to the slowing down the fault motion component due to tectonic forces. Due to the small size of the fault crossing, as the trend component with the same strain rate was recorded even before dam construction and lake filling, we attribute the long-term strain component to the slow regional tectonic stress action. The results of half a century of permanent monitoring of the deformation of the fault zone, which crosses the dam foundation, has led to the understanding of the complicated dynamics of the fault zone deformation, reflecting a joint influence of local tectonics, man-made engineering stresses, and environmental factors.

## Data Availability

Data Availability Statement: No new data were created of analyzed in this study.

## References

[1] Chelidze T., Matcharashvili T., Abashidze V., Tsaguria T., Dovgal N., Zhukova N. (2019). Complex dynamics of fault zone deformation under large dam at various time scales. Geomech. Geophys. Geo-Energy Geo-Resour..

[2] Chelidze T., Matcharashvili T., Mepharidze E., Dovgal N. (2023). Complexity in Geophysical Time Series of Strain/Fracture at Laboratory and Large Dam Scales. Entropy.

[3] Chelidze T., Sborshchikovi A., Mepharidze E. (2024). Nonlinear Analysis of the Enguri Dam Geodynamical Datasets. Bull. Georgian Natl. Acad. Sci..

[4] Hurst H.E. (1951). Long-term storage capacity of reservoirs. Trans. Am. Soc. Civ. Eng..

[5] Hubbert M., Rubbey W. (1959). Role of fluid pressure in mechanics of overthrust faulting. Bull. Geol. Soc. Am..

[6] Peinke J., Matcharashvili T., Chelidze T., Gogiashvili J., Nawroth A., Lursmanashvili O., Javakhishvili Z. (2006). Influence of periodic variations in water level on regional seismic activity around a large reservoir. Phys. Earth Planet. Inter..

[7] Bartsh M., Schiess Z., Steiger K. (2011). Continuous dam monitoring: An essential basis for reliable back-analysis. Hydropower Dams Int. J..

[8] Matcharashvili T., Chelidze T., Abashidze V., Zhukova N., Fra Paleo U. (2011). Evidence for changes in the dynamics of Earth crust tilts caused by the large dam construction and reservoir filling at the Enguri dam international test area (Georgia). Nonlinear Dyn..

[9] Roeloffs E. (1988). Fault stability changes induced beneath a reservoir with cyclic variations in water level. J. Geophys. Res. Solid Earth.

[10] Zhang S., Zheng D., Liu Y. (2022). Deformation Prediction System of Concrete Dam Based on IVM-SCSO-RF. Water.

[11] Aghajanzadeh S., Ghaemian M. (2013). Nonlinear Dynamic Analysis of Concrete Gravity Dam Considering Elasto-plastic Constitutive Model for Foundation. Sci. Iran..

[12] Lyu X., Yang K., Xu C., Fang J., Duan M., Zhang Z. (2024). Experimental study of mechanical properties of artificial dam for coal mine underground reservoir under cyclic loading and unloading. Geomech. Geophys. Geo-Energy Geo-Resour..

[13] Kantz H., Schreiber T. (1997). Nonlinear Time Series Analysis.

[14] Kwak N., Choi C.H. (2002). Input Feature Selection for Classification Problems. IEEE Trans. Neural Netw..

[15] Peng H., Long F., Ding C. (2005). Feature selection based on mutual information criteria of max-dependency, max-relevance, and min-redundancy. IEEE Trans. Pattern Anal. Mach. Intell..

[16] Schuster H. (2010). Reviews of Nonlinear Dynamics and Complexity.

[17] Strogatz S.H. (2000). Nonlinear Dynamics and Chaos.

[18] Butte A.J., Kohane I.S. Mutual information relevance networks: Functional genomic clustering using pairwise entropy measurements. Proceedings of the Pacific Symposium.

[19] Li W. (1990). Mutual information functions versus correlation functions. J. Stat. Phys..

[20] Press W., Teukolsky S., Vetterling W., Flannery B. (2006). Numerical Recipes: The Art of Scientific Computing. Conditional Entropy and Mutual Information.

[21] Tsallis C. (1988). Possible generalization of Boltzmann-Gibbs statistics. J. Stat. Phys..

[22] Tsallis C. (2009). Introduction to Nonextensive Statistical Mechanics: Approaching a Complex World.

[23] Baratpour S., Khammar A. (2016). Results on Tsallis entropy of order statistics and record values. Istat. J. Turk. Stat. Assoc..

[24] Cover T.M., Thomas J.A. (2005). Elements of Information Theory.

